# Issues in localization of brain function: The case of lateralized frontal cortex in cognition, emotion, and psychopathology

**DOI:** 10.3389/fnint.2013.00002

**Published:** 2013-01-30

**Authors:** Gregory A. Miller, Laura D. Crocker, Jeffrey M. Spielberg, Zachary P. Infantolino, Wendy Heller

**Affiliations:** ^1^Department of Psychology, University of DelawareNewark, DE, USA; ^2^Department of Psychology, University of Illinois at Urbana-ChampaignChampaign, IL, USA; ^3^Zukunftskolleg, University of KonstanzKonstanz, Germany; ^4^Department of Psychology, University of CaliforniaBerkeley, CA, USA

**Keywords:** emotion, motivation, frontal cortex, lateralization, localization

## Abstract

The appeal of simple, sweeping portraits of large-scale brain mechanisms relevant to psychological phenomena competes with a rich, complex research base. As a prominent example, two views of frontal brain organization have emphasized dichotomous lateralization as a function of either emotional valence (positive/negative) or approach/avoidance motivation. Compelling findings support each. The literature has struggled to choose between them for three decades, without success. Both views are proving untenable as comprehensive models. Evidence of other frontal lateralizations, involving distinctions among dimensions of depression and anxiety, make a dichotomous view even more problematic. Recent evidence indicates that positive valence and approach motivation are associated with different areas in the left-hemisphere. Findings that appear contradictory at the level of frontal lobes as the units of analysis can be accommodated because hemodynamic and electromagnetic neuroimaging studies suggest considerable functional differentiation, in specialization and activation, of subregions of frontal cortex, including their connectivity to each other and to other regions. Such findings contribute to a more nuanced understanding of functional localization that accommodates aspects of multiple theoretical perspectives.

Across decades of research to identify the functions served by various brain regions, using non-invasive, low-density scalp EEG recording, emphasis on large brain regions was understandable, because more localized inferences were rarely feasible. Proposals about broad functional differences in the cerebral hemispheres were common. A prominent literature in that tradition attempted to assign functions differentially to left- and right-frontal or posterior cortex, and much attention was spent on frontal lateralization and frontal specialization with respect to emotion and emotion-related psychopathology such as depression.

The emotion literature more generally has yet to settle on a dominant set of concepts for mapping emotion phenomena, with various definitions of and time courses for emotion, affect, mood, and motivation in use (e.g., Gendron and Barrett, 2009; Lindquist et al., 2013). For example, central or peripheral physiology associated with emotion is commonly treated as a *response to* emotion, but some have proposed that the physiology is *part of* emotion (e.g., Lang, 1979; Niedenthal, 2007). Thus, what it means to “have” an emotion is, in part, having the relevant physiology. Definitions as well as relevant psychological and biological mechanisms overlap for emotion, motivation, etc. The diversity of conceptualizations of emotion adds methodological and interpretive variance to the literature on frontal lateralization of function.

## Two models of frontal lateralization

The present review contends that key assumptions in the debate about frontal lateralization are untenable in light of recent research on frontal cortex. A longstanding literature has argued that frontal differences in activation track emotional valence or mood, with left-frontal activation associated with positive stimuli or mood and right-frontal activation associated with negative stimuli or mood (e.g., Heller and Levy, 1981; Tucker, 1981; Davidson, 1983, 1984, 1992; Heller, 1990; Heller et al., 1998). A growing literature has argued for a different interpretation of lateralized frontal activity, with approach and avoidance (or the closely related concept of withdrawal) motivation as the relevant dichotomy (for reviews, see Davidson, 2003; Harmon-Jones, 2003; Harmon-Jones et al., 2010). Davidson (1983) proposed that frontal asymmetry is not related fundamentally to the valence of an emotional stimulus but to the motivational system that is engaged by that stimulus. He posited that left prefrontal cortex (PFC) is involved in a system facilitating approach to appetitive stimuli and right PFC in a system facilitating avoidance of aversive stimuli. In this model, it is not processing related to emotional valence itself that is lateralized in PFC. Rather, emotion-related lateralization is observed because emotions involve approach and/or avoidance components. Therefore, emotion will be associated with a left or right lateralization depending on the extent to which it is accompanied by approach or avoidance motivation (Davidson, 1983). Several related dichotomies have been proposed, including Dickinson and Dearing's (1979) Aversive/Attractive systems, Gray's (1994) Behavioral Activation/Behavioral Inhibition systems, and Lang et al.'s (1990) Appetitive/Defensive systems (for reviews, see Lang et al., 1990; Davidson and Irwin, 1999; Elliot and Covington, 2001).

Wacker et al. (2003) noted that the valence perspective on frontal asymmetry had persisted for two decades with very little direct examination of whether related constructs such as motivation or behavioral activation/inhibition would do as well or better. More recently the literature has attempted to choose between those interpretations (e.g., Spielberg et al., 2008; Carver and Harmon-Jones, 2009b; Herrington et al., 2009). Here it is argued that no such choice is needed, if a finer degree of cortical granularity is considered.

## Evolving conceptualizations of the functional role of EEG alpha

Much of the evidence that forms the foundation of these two traditional views of lateralized function rests on EEG studies of hemispheric asymmetries in alpha-band activity. This research has long relied on the view that alpha-band activity is inversely related to the level of nearby regional brain activity. Besides some well-known methodological challenges (Allen et al., 2004), there are substantive challenges to this traditional view, beginning with the functional role of alpha. Rather than being simply a non-specific index of regional activity, alpha and other low-frequency oscillations foster communication between brain regions, whereas high-frequency oscillations facilitate coordination within cell assemblies on a much smaller scale (Kopell et al., 2000; von Stein and Sarnthein, 2000). Klimesch et al. (2007) argued that reduced alpha facilitates relatively unfiltered throughput, whereas increased alpha facilitates processing of specific features of a current stimulus or of an accessed memory relevant to the current task or goal state. Furthermore, cross-frequency coupling (frequency-specific, correlated oscillations in distributed networks) has been proposed as an index of network interaction across brain regions (Siegel et al., 2012). These distinct distant/local roles can converge, for example when there is cross-frequency coupling between alpha phase and gamma amplitude, in the form of alpha driven by region X modulating gamma in region Y. This so-called phase-amplitude coupling can be quantified as a phase-locking value relating the phase of activity in one frequency band to the amplitude of activity in another (typically higher) frequency band (Lachaux et al., 1999). Voytek et al. (2010, p. 191) identified phase-amplitude coupling as reflecting a “… means through which multiple overlapping long-range networks can communicate by statistically biasing the extracellular membrane potential in local cortical regions such that neurons will be more likely to fire during particular phases or phase network ensembles of low-frequency oscillations.”

This richer and more functionally specific perspective on alpha activity provides a means to re-examine longstanding assumptions as well as to develop new predictions about cognition-control regions modulating activity in emotion-control regions (at least to the extent that cognition and emotion can be distinguished; Miller, 1996; Pessoa, 2005; Duncan and Barrett, 2007; Mohanty et al., 2007; Dolcos et al., 2011). For example, region-specific correlations between alpha phase and gamma amplitude might be observed in regions related to emotion regulation, given that alpha oscillations modulate the state of sensory brain regions to direct the flow of information and optimize performance (Jensen and Mazaheri, 2010; White et al., 2010; Hanslmayr et al., 2011; Popov et al., 2012a,b) and that large-scale cortical interactions in the alpha/beta range influence gamma activity (Siegel et al., 2012). Thus, although EEG alpha conceived as a nonspecific activity metric has been the dominant tool in the debate over valence/arousal and approach/avoidance constructs regarding frontal lateralization, it provides a problematic foundation on which to base conclusions about regional brain organization.

## Lateralization in frontal lobes as the unit of analysis and of conceptualization

A second substantive challenge to the traditional use of alpha in studies of frontal lateralization is that the notion of “brain region” is problematic in this context, such as when two sets of neurons are treated as anatomically and functionally quite distinct (and the neurons within a “region” are treated as functionally homogenous). Consensus has not been reached on how to segment even the gross structural or functional anatomy of the brain, particularly in the face of individual differences (Brett et al., 2002). Although the psychological functions served by specific regions cannot themselves be anatomically localized (Miller, 2010; see also Lindquist et al., 2013), there is considerable momentum to view demarcated brain regions as serving or implementing distinct, specific functions. Granting that such an oversimplification can be an appropriate methodological expedient, a question of granularity arises: how big (small) a region to treat as a functional unit?

Surely fruitful answers to the granularity question will vary by psychological function, by brain region, and by research method. The extensive literature on functional laterality in frontal cortex has commonly treated the left- and right-frontal lobes as the units of analysis, and the amount of EEG alpha recorded over them has typically, as reviewed above, been interpreted as an (inverse) index of neural activity in those units. Accordingly, differences in EEG recorded over left- and right-frontal cortex have been used to infer lateralized specialization or function. The following discussion examines a particular line of research by the authors and their colleagues for evidence about the appropriate granularity for the literature on frontal lateralization in emotion and suggests that hemisphere-level models of functional differentiation are no longer viable.

## Practical challenges to hemisphere-level models

As noted above, implicitly and sometimes explicitly the literatures arguing for valence or approach interpretations have often treated the left- and right-frontal lobes as single functional units. This assumption was understandable given that, until recently, much of the research involved scalp EEG studies relying on what are now considered low-density montages or, less commonly, lesion patients with uncertain or inconsistent trauma. Dense-array EEG recording montages can provide more precise localization of activity. However, the frontal lobes pose particular challenges to EEG source localization, especially in the absence of hypotheses about specific, dipolar sources, typically leading to reliance on the relatively low spatial localization precision of methods aimed at identifying distributed sources. Many studies were undertaken without individual structural MRI (which would allow for individual differences in brain structure) and before dense-array EEG recording was widely available. EEG source analysis was rarely attempted, and inferences beyond the level of hemisphere, cortical quadrants, or gross superior/inferior distinctions were rarely advanced.

The frontal-laterality EEG literature faces additional challenges, including reliance on resting data with little knowledge of or control over what subjects are doing as well as methodological disputes about choice of reference site, inter-session replicability, and laterality quantification metrics (e.g., Allen et al., 2004; Davidson, 2004). Davidson (2004) suggested that the frontal cortex is a large territory with considerable, if controversial, functional differentiation. Considerable research addressing the valence/motivation dispute, with much better localization of findings, has subsequently accrued. On balance it provides partial support to both views, in that different regions of frontal cortex are associated both with different functions and with functionally different networks of brain regions. Even a very selective review of recent fMRI studies demonstrates quite diverse functions associated with different regions of frontal cortex. The present review draws on a program of research using color-word and emotion-word variants of the Stroop task, not only to limit the scope of the review but to demonstrate that support for diverse functions and diverse localizations can be observed even within a single task in a single line of study.

## Experimental challenges to hemisphere-level models

**Figure 1:**
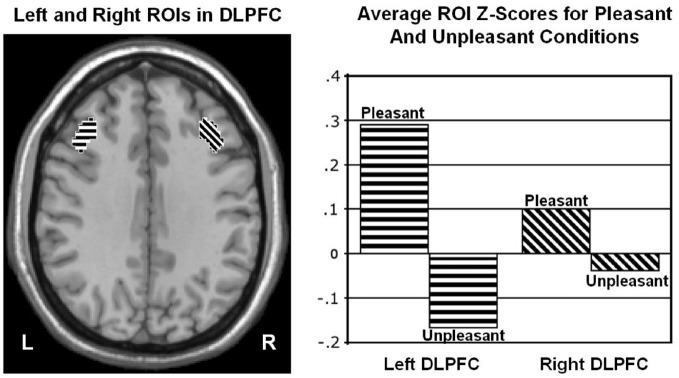
**Region yielding a Valence × Hemisphere interaction in middle frontal gyrus of dorsolateral prefrontal cortex (DLPFC). Left panel**: Regions of interest (ROIs) used to quantify activity in left (L) and right (R) dorsolateral prefrontal cortex at Talairach *z* = 34 mm. **Right panel**: Mean *z* scores for pleasant and unpleasant word conditions in left and right DLPFC. From Herrington et al. (2005). Copyright by the American Psychological Association. Reprinted with permission.

**Figure 2:**
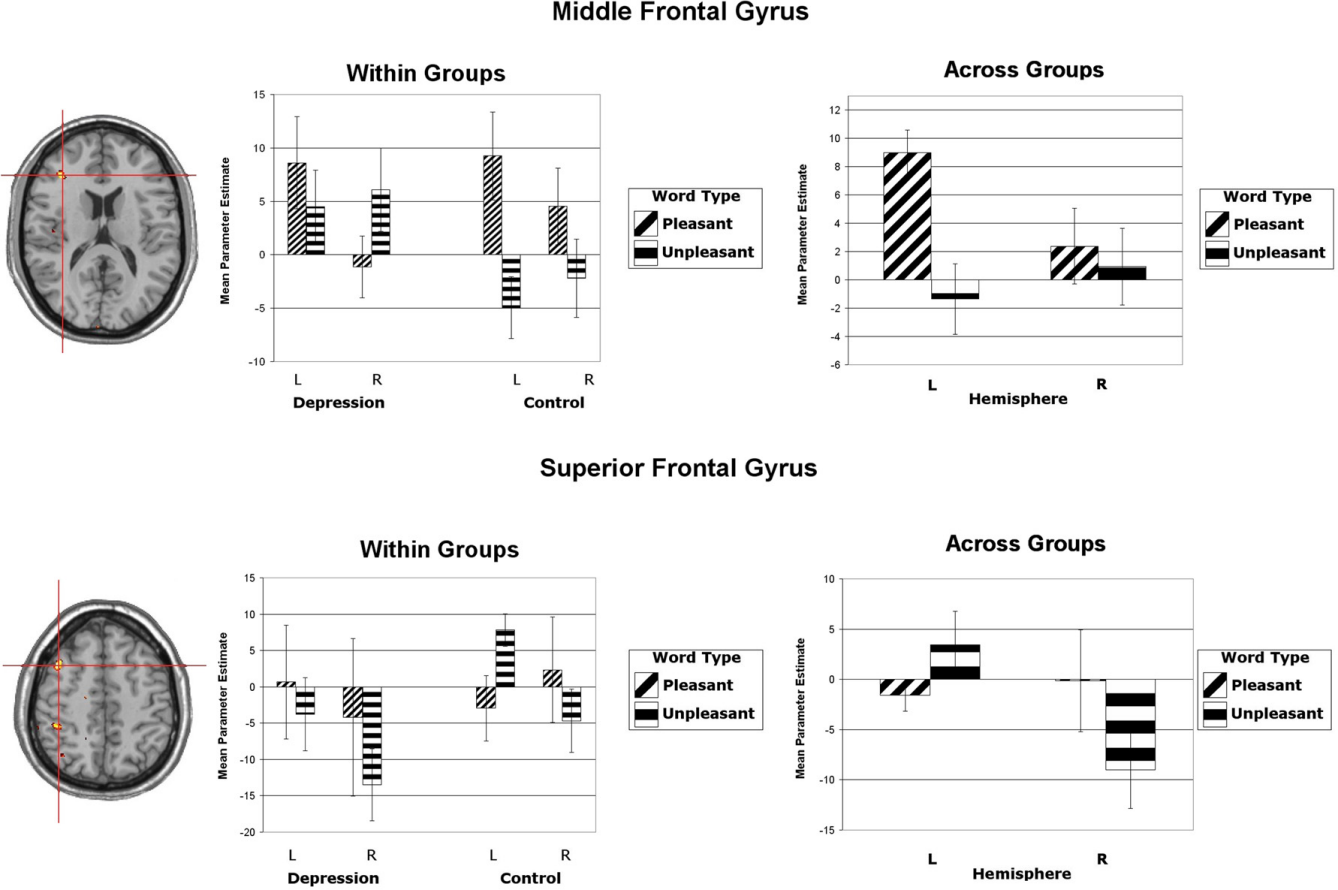
**Upper panels:** Region yielding a Valence × Hemisphere interaction in middle frontal gyrus of dorsolateral prefrontal cortex. The left panel shows the significant cluster in an axial slice at Talairach *z* = 16. (Although the center of this cluster was inferior to that illustrated in Figure 1, the means showed the same pattern.) **Lower panels:** Region yielding a Valence × Hemisphere interaction in superior frontal gyrus of dorsolateral prefrontal cortex. The left panel shows the significant cluster in an axial slice at Talairach *z* = 49. **All panels**: Activation is arbitrarily overlaid on left-hemisphere anatomy, as hemisphere was included as a factor in the analysis. The red crosshairs are placed over the center of effect size. Bar graphs are mean parameter estimates for each level as a function of Valence and Hemisphere by group and averaged across groups. Error bars represent 1 standard error above and below the mean. From Herrington et al. (2010). Copyright the Society for Psychophysiological Research.

**Figure 3:**
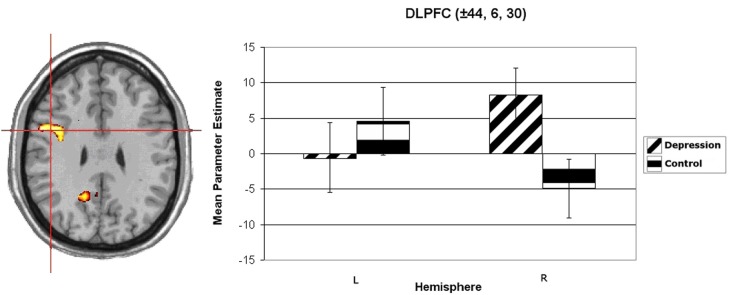
**Group × Hemisphere interaction for unpleasant vs. neutral word activation in dorsolateral prefrontal cortex (DLPFC).** Activation is arbitrarily overlaid on left-hemisphere anatomy, as hemisphere was included as a factor in the analysis. The red crosshairs are placed over the center of effect size for the cluster. Panel on right plots average parameter estimates as a function of Group (Depression, Control) and Hemisphere. Coordinates are the center of effect size at *z* = 30 in Talairach space. Error bars represent 1 standard error above and below the mean. From Herrington et al. (2010). Copyright the Society for Psychophysiological Research.

Wager et al. (2003) reviewed hemodynamic neuroimaging studies of emotion, finding little support for valence-specific lateralization of emotion, including in frontal cortex. However, Herrington et al. (2009) noted that inclusion of a hemisphere factor in analyses is remarkably rare in the fMRI literature, even though it is often essential when making claims about lateralization. Herrington et al. (2005) provided the first fMRI demonstration of left-frontal lateralization associated with positive valence. As illustrated in Figure 1, Herrington et al. (2005) reported both Valence and Valence × Hemisphere effects for an empirically defined region of interest (ROI) in left vs. right dorsolateral prefrontal cortex (DLPFC). Using an independent sample, Herrington et al. (2010) replicated this finding (Figure 2, upper panels). Herrington et al. (2010) identified a separate DLPFC region that showed enhanced rightward lateralization in depression (Figure 3). These fMRI studies thus support earlier EEG literature drawing the same conclusions—positive valence associated with left-frontal activation and depression associated with right-frontal activation. However, Herrington et al. (2010) found another area of DLPFC showing a contrary lateralization, with response to negative words more left-lateralized (Figure 2, lower panels). Such results suggest that a hemisphere- or cortical-quadrant-level view of frontal lateralization is inadequate.

**Figure 4:**
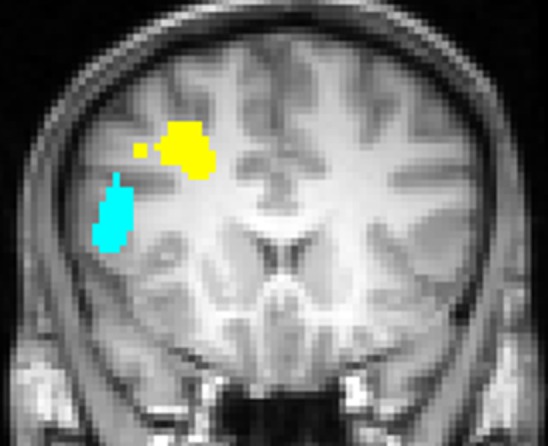
**Left inferior frontal gyrus (blue) region more active for negative words than for neutral words, significantly moreso for subjects scoring high in anxious apprehension, and left dorsolateral prefrontal cortex (yellow) region more active for positive words than for neutral words, with no differentiation by level of anxious apprehension or anxious arousal, displayed at Talairach *y* = 18.** Neurological convention (left-hemisphere on left of panel). From Engels et al. (2007). Copyright the Society for Psychophysiological Research.

Engels et al. (2007) replicated the leftward lateralization in DLPFC associated with positive stimuli. In addition, they identified a distinct, non-overlapping left inferior frontal gyrus (IFG) ROI that was sensitive to worry/anxious apprehension (Figure 4). The determination that anxious apprehension and positive valence are associated with distinct left PFC regions puts to rest an apparent contradiction that traditional, low-density EEG studies (e.g., Heller et al., 1997; Nitschke et al., 1999) could not address, that a negative emotion (worry) seemed to be localized to the same brain quadrant as a positive emotion: frontal cortex is functionally differentiated with respect to emotion processing.

Engels et al. (2010) replicated the rightward lateralization of DLPFC activity in depression, showing it to depend on a particular pattern of comorbid anxiety (high anxious arousal). In contrast, Engels et al. (2010) found reduced rightward lateralization in a separate region, in IFG, again moderated by comorbid anxiety (high anxious arousal, low anxious apprehension). Thus, even within a hemisphere, frontal areas can show contrasting relationships with psychological variables, some of which are consistent with the traditional valence interpretation of frontal lateralization, and some of which are not. Furthermore, when anxious arousal is high and anxious apprehension is low, depression is associated with a decrement in left DLPFC (Engels et al., 2010). High anxious apprehension appears to counteract this pattern, possibly by boosting brain activity in compensatory regions of left PFC. The findings indicate that, if both types of anxiety are not taken into account, activation asymmetries for depression may not be reliably detected, yet another contribution to the lack of consistency in the literature. It is easy to imagine that a literature employing various tasks, and involving a variety of psychological functions differentially engaging various brain regions, could produce diverse findings.

**Figure 5:**
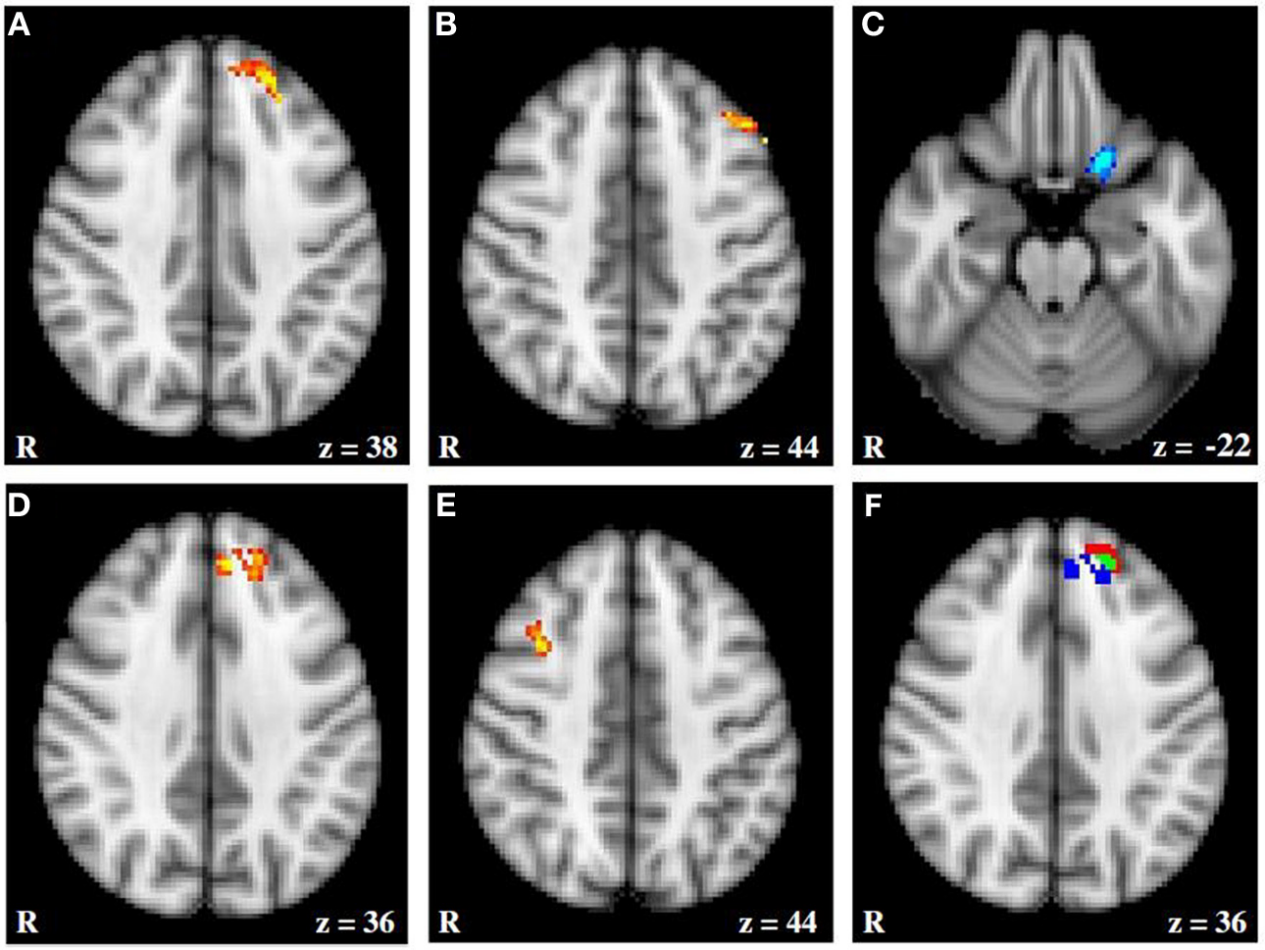
**fMRI activation moderated by approach and avoidance temperament. (A** and **B**) Activation for Incongruent–Congruent contrast (IvC) correlating positively with approach temperament. **(C)** Activation for IvC correlating negatively with approach temperament. (**D** and **E**) Activation for IvC correlating positively with avoidance temperament. **(F)** Overlap between activation correlating with approach and avoidance temperament; red = activation associated with approach; blue = activation associated with avoidance; green = overlap in activation. Axial slices are at MNI 152 *z* coordinates noted. From Spielberg et al. (2011). Copyright Elsevier Inc.

This line of fMRI studies finding evidence of frontal lateralization related to valence also examined activation associated with approach and avoidance concepts. As illustrated in Figure 5, Spielberg et al. (2011) described two left-hemisphere DLPFC areas in which incongruent color-words prompted more activity than did congruent words in subjects with high scores on self-report measures of approach temperament. However, a left medial-posterior orbital frontal cortex region showed the opposite effect. This contrary lateralization, in line with other studies of orbitofrontal cortex and emotional valence (for reviews, see O'Doherty, 2004; Wager et al., 2008), could also contribute to inconsistencies in the traditional EEG alpha literature. Dolcos et al. (2011) reviewed studies showing diverse functional connectivity between various regions of frontal cortex and limbic regions. These could contribute to different regions of frontal cortex having different roles in emotion processing and showing different functional lateralization.

**Figure 6:**
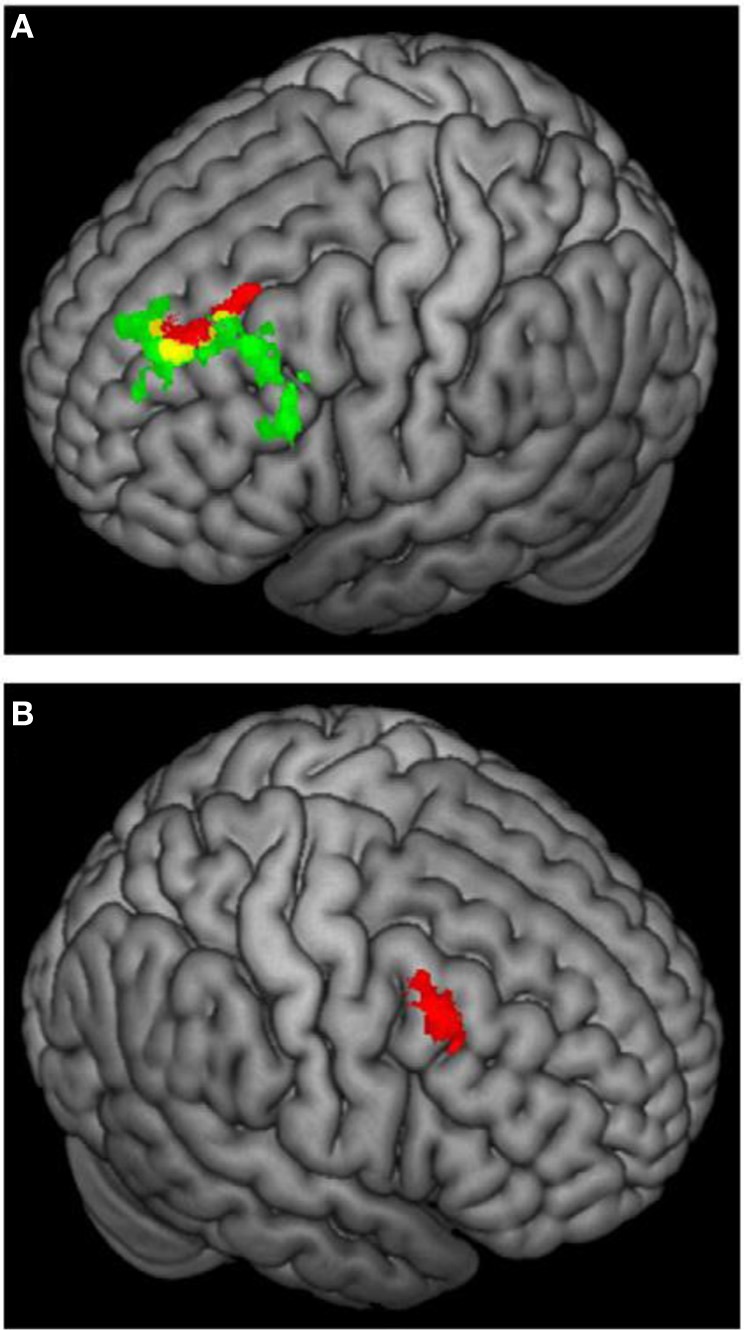
**3D rendering of fMRI activation moderated by approach and avoidance temperament. (A)** Activation for incongruent vs. congruent contrast correlating positively with approach temperament (green) or positively with avoidance temperament (red); yellow = overlap in activation. **(B)** Activation for incongruent vs. congruent contrast correlating positively with avoidance temperament. From Spielberg et al. (2011). Copyright Elsevier Inc.

Figure 6 (Spielberg et al., 2011) illustrates that, close to a left-hemisphere frontal region showing the predicted positive relationship with approach temperament, a left-hemisphere region showed a positive relationship with avoidance temperament, contrary to traditional prediction. (The approach-related cluster was significantly lateralized, whereas the avoidance-related cluster was not.) Indeed, there was some overlap, such that activation in an 18-voxel subregion correlated positively with both approach and avoidance temperament. Spielberg et al. (2012b) replicated the association between approach motivation and left-hemisphere activation in lateralized regions of DLPFC and also of avoidance and right DLPFC. However, these effects were not moderated by the valence of the stimuli, further evidence that processes serving motivation and emotional valence can be implemented in different regions of PFC. Furthermore, the left-frontal regions sensitive to approach motivation, positive valence, and anxious apprehension were mutually distinct. These findings again indicate that frontal cortex is functionally differentiated in a way that belies gross regional generalizations.

Studies of psychopathology in this line of research further underscore the diversity of frontal-lobe specialization. Recent work (e.g., Figure 3) has identified a brain region in left PFC that points to a mechanism by which depression may interfere with the ability to modulate top–down attentional processing, degrading concentration and task performance (Levin et al., 2007; Engels et al., 2010; Herrington et al., 2010). Furthermore, different types of anxiety (Nitschke et al., 1999, 2001) modulate PFC activity in distinct ways. As noted above, a left PFC brain region more active when anxious apprehension is high, distinct from a left PFC region active in a positive valence context (differentiated in Figure 4), contrasts with a right-hemisphere region more active when anxious arousal is high (Engels et al., 2007). Thus, “anxiety” is not a monolithic phenomenon whose cortical instantiation can be assigned to a single brain region, and accordingly it does not show a single, consistent pattern of lateralization.

## Anger: a decisive testing ground?

A potentially informative manipulation in the literature on emotion and frontal lateralization has involved anger. Harmon-Jones (2003) and others have noted that anger is typically classified as involving both approach and negative valence, so it seems uniquely useful in comparing valence/arousal and approach/avoidance interpretations, which otherwise tend to face methodological confounds (see Carver and Harmon-Jones, 2009a,b, for review and response to commentaries). In commentaries on that review, Watson (2009) raised concerns about this strategy, arguing that anger shows both approach and avoidance properties (see also Stewart et al., 2010), and Tomarken and Zald (2009) suggested that hemodynamic neuroimaging results then available generally did not support the approach interpretation of frontal laterality (though see Herrington et al., 2009; Berkman and Lieberman, 2010).

Harmon-Jones (2004) developed a self-report questionnaire to assess subjects' attitude toward anger, documenting that anger can be judged to be a positive feeling but that this did not account for EEG alpha results indicating leftward frontal laterality associated with trait anger. Stewart et al. (2008) went further, noting that anger may sometimes have important positive valence or appetitive qualities rather than being exclusively negative in valence, so anger manipulations may not unambiguously distinguish valence and approach views. Focusing on resting alpha asymmetry, Stewart et al. (2008) demonstrated that the anger/asymmetry story for EEG alpha is complex, with different anger styles (anger-out vs. anger-in) showing distinct lateralization patterns. Furthermore, anxious apprehension moderated anger-related lateralization. In addition, subjects high in trait anger who differed in approach- and avoidance-related motivational tendencies displayed greater left-frontal lateralization than did control participants regardless of motivational direction. These results are not well explained by either valence or motivation views. Partly because anger is multifaceted, it has not proved to be the decisive context for resolving the valence/motivation dispute that was hoped for.

## Two models of frontal lateralization: current status

The present selective review has emphasized a single line of research from a single lab. Although this limits generalizability, it has the value of holding constant a host of variables that normally confound comparisons of findings across studies. The body of work reviewed here shows that, even holding many things constant, in a single lab, most of it on a single MRI scanner, considerable, systematic, and replicable regional differentiation of lateralized frontal function is apparent, associated with a variety of psychological constructs.

On the debate between the valence/arousal interpretation and the approach/avoidance interpretation of frontal lateralization of emotion, the literature provides numerous examples of support for each, recently replicated in the fMRI studies discussed above. But contrary findings and caveats also abound. The frontal cortex is a large landscape with enormous potential regional specialization that need not follow a simple theme (e.g., Brodmann Area 10 alone may have numerous, differentially specialized subregions; Burgess et al., 2011). Attempts to choose between a general valence/arousal account and a general approach/avoidance account of lateralized activity associated with emotion, motivation, or psychopathology no longer seem viable. The present contention is that the literature now makes clear that neither account is of much help in providing a comprehensive account of frontal function, lateralized or otherwise.

It may be tempting to retain longstanding approaches as long as no equally comprehensive replacement is available, and it can be noted that individual differences may have complicated interpretation of particular findings that challenge those approaches. It can also be argued that neither model has been thoroughly tested. But enough conceptual, practical, and experimental challenges have accrued that neither traditional approach seems viable. The present review suggests that the debate between those two positions, while historically generative, should be over. Both may be still useful in specific contexts, but both now appear too coarse, and neither is comprehensive.

On the issue of EEG as a means of addressing such questions, a focus on large brain regions was useful across decades of low-density scalp EEG recording. In modern-day EEG research, low density often still suffices for some purposes. For example, some components of the event-related brain potential with sufficiently distinctive and well established topography, with well-known sensitivity to parameters such as age, stimulus modality, and task, may be measured effectively with just a few recording sites. When distinctions are less established, or when spatial localization is important in identifying phenomena, higher-density recording can be invaluable, especially if augmented with MEG or MRI (e.g., Silton et al., 2010, 2011; White et al., 2010; Hanlon et al., 2011; Williams et al., 2011).

More is often better, but even with high-density recording (generally considered to be 64+ channels) one faces choices. One cannot surround the head with sites. There are times when local electrode density may be more important, such as distinguishing finger locations in motor cortex, and times when spatial extent may be more important, such as localizing deeper sources (e.g., Hanlon et al., 2011; Williams et al., 2011). In the design of an electrode montage for general-purpose use, a critical choice, other than number of channels, is how inferior to place the most inferior electrodes. Reaching to or beyond the cheekbone and below the eyes can substantially enhance representation of more inferior frontal brain activity, at some cost of increased artifact. Depending on what one is trying to study, where one positions the electrodes, and a host of other trade-offs, EEG may achieve sub-centimeter source localization accuracy, better than routine fMRI and better than needed for many purposes (Miller et al., 2007; Aine et al., 2012). Dense-array MEG can often do somewhat better still. fMRI optimized for such localization can do even better, at some cost to temporal resolution, though trade-offs can provide improved temporal resolution as well. Rather than cast various neuroimaging methods as competing, it is their complementarity and integration that will benefit the field.

It has become clear that traditional hemisphere and quadrant models of regional brain function in emotion and psychopathology are generally too coarse. Network accounts of brain function are growing in prominence, though challenging to operationalize and test. For example, Spielberg et al. (2012a) offered a proposal for a network in frontal cortex and other areas subserving motivation that accommodates many of the findings reviewed above. Sheline et al. (2010) proposed that a region of dorsal medial PFC serves as a critical junction for three resting-state networks reaching beyond frontal cortex and found that this region shows exaggerated connectivity to those networks in depression. The optimal level of granularity will surely vary widely, as a function of research context. Regional specialization may even be adaptive and thus beneficially unstable (Duncan, 2001) on a variety of temporal and spatial scales. Much good work lies ahead, with the proviso that localization is of brain activity, not psychological function, and that the psychological and biological phenomena we pursue need to be understood across multiple scales in parallel.

### Conflict of interest statement

The authors declare that the research was conducted in the absence of any commercial or financial relationships that could be construed as a potential conflict of interest.
